# Role of MHC-Linked Susceptibility Genes in the Pathogenesis of Human and Murine Lupus

**DOI:** 10.1155/2012/584374

**Published:** 2012-06-19

**Authors:** Manfred Relle, Andreas Schwarting

**Affiliations:** First Department of Medicine, University Medical Center of Johannes Gutenberg University of Mainz, 55131 Mainz, Germany

## Abstract

Systemic lupus erythematosus (SLE) is a chronic autoimmune disease characterized by the production of autoantibodies against nuclear antigens and a systemic inflammation that can damage a broad spectrum of organs. SLE patients suffer from a wide variety of symptoms, which can affect virtually almost any tissue. As lupus is difficult to diagnose, the worldwide prevalence of SLE can only be roughly estimated to range from 10 and 200 cases per 100,000 individuals with dramatic differences depending on gender, ethnicity, and location. Although the treatment of this disease has been significantly ameliorated by new therapies, improved conventional drug therapy options, and a trained expert eye, the underlying pathogenesis of lupus still remain widely unknown. The complex etiology reflects the complex genetic background of the disease, which is also not well understood yet. However, in the past few years advances in lupus genetics have been made, notably with the publication of genome-wide association studies (GWAS) in humans and the identification of susceptibility genes and loci in mice. This paper reviews the role of MHC-linked susceptibility genes in the pathogenesis of systemic lupus erythematosus.

## 1. Introduction

Chronic autoimmune diseases have complex pathogeneses and the course of events leading to these diseases is not well understood. They arise from a dysfunction of the immune system, recognizing self-antigens as foreign, which can lead to inflammation and severe damage of tissues and organs. One of these complex inflammatory diseases is called systemic lupus erythematosus (SLE). The etiology of lupus is multifactorial with environmental, hormonal, ethnic, and genetic factors [1].

In the 70s and 80s of the last century mouse models of spontaneous lupus, like (NZB × NZW) F1 hybrids, BXSB mice (which carry the disease-accelerating *Yaa* gene on the Y chromosome [2–4]), MRL/*lpr* mice (MRL mice homozygous for a *fas* mutation [5, 6]) or MRL/*gld* mice (MRL mice homozygous for a *fasL* mutation [7, 8]) were established [9–12]. Research based upon these mice revealed that a number of genes, loci, and pathways are directly associated with lupus in both mouse and human species (reviewed in [13–17]). In addition, by means of these models signaling pathways were identified that are dysregulated in both human and murine lupus. Hence, mouse models will continue to serve as invaluable instruments for studying the genetic basis of lupus susceptibility, because they depict the genetic facets of the human systemic lupus erythematosus (SLE).

Recent findings suggest that aberrant epigenetic mechanisms may be involved in the pathogenesis of lupus [18], and a number of genes have been claimed to be targets of these alterations [19]. However, the mechanisms underlying epigenetic changes are poorly understood. Deciphering the contribution of epigenetic alterations to the pathogenesis of lupus will provide promising insights in this complex autoimmune disease and epigenetic pharmaceuticals will offer new therapeutic options to treat SLE.

One of the genetic risk factors for the development of lupus (or other immune-mediated diseases) are genes linked to the major histocompatibility complex (MHC) [20]. In humans, HLA antigens have long been associated with SLE and, therefore, these susceptibility genes are extensively studied [21]. Certain HLA class II genes or haplotypes seem to be particularly involved on lupus pathogenesis [14, 22–24]. HLA class III genes, such as those encoding the complement components C2 and C4, may also be considered as risk factors for the development of a lupus-like disease in different ethnicities [25]. In mice, it could also be shown that the MHC class II locus directly participates in lupus disease susceptibility similar to that observed in humans [26]. The effect of MHC-linked complement factors on disease expression is strongly dependent on the background genes, reflecting the genetic unification of inbred mice in comparison to wildtype mice.

However, the role of certain MHC haplotypes, genes, or alleles in lupus pathogenesis is still controversially discussed. For this reason and to update the most recent scientific research on this topic, this paper reviews the role of MHC genes and alleles in the pathogenesis of both human and murine lupus.

## 2. The Major Histocompatibility Complex (MHC)

### 2.1. Historical Overview

More than a century ago, it was observed that tissue transplants (now called allografts) of one animal were rejected when transferred to a different laboratory mouse. At the Jackson Laboratory Gorer showed in 1937 that so-called H or “histocompatibility antigens” on the surface of mouse cells account for this [27, 28]. Seven years later, it was Medawar who showed that allograft rejection is a host versus graft reaction [29, 30]. At the same time, Snell developed congenic mice strains that were genetically identical except at the H-2 locus. With the aid of these mice he could show that the H-2 antigens were “controlled” by genes at the H-2 complex on chromosome 17 and called this multigene locus “major histocompatibility complex” (MHC) [31–33]. In 1958, the first human alloantigen present on leucocytes was detected by Dausset, which was later called HLA-A2 [34, 35]. A few years later Payne and coworkers depicted the first human multiallelic system, now known as the HLA class I loci HLA-A and HLA-B [36]. However, it was clear from the beginning that allograft rejection or acceptance is not the physiological function of MHC molecules. In the early sixties, experiments of Benacerraf et al. with guinea pigs and synthetic amino acid polymers showed that there is a single genetic locus which controls the immune system's ability to respond to foreign antigens and called the (autosomal dominant) genes of this locus “immune response genes” (or Ir genes) [37–39]. In the late 1960's, McDevitt found that the Ir genes were linked to the MHC [40, 41]. The concept of immune response genes was refined by Zinkernagel and Doherty (in 1974), who made the breakthrough discovery that the ability of virus-specific T lymphocytes to combat a virus infection is dependent upon the simultaneous recognition of both “foreign” molecules of the virus and self molecules (i.e., major histocompatibility proteins) [42]. This limitation or narrowing of antigen recognition by T cells was called “MHC-restricted antigen recognition” or in short, “MHC restriction” and was subsequently confirmed in many other systems. One year before Zinkernagel and Doherty made their pioneering discovery, the first disease-associated MHC allele, namely, HLA-B27, was reported. HLA-B27 is strongly associated with ankylosing spondylitis [43, 44].

### 2.2. Genetics of HLA and H-2

The major histocompatibility complex is located on the short arm of chromosome 6 in humans and on the telocentric chromosome 17 in mice [45, 46]. The genes coding for the classical transplantation antigens as well as the so-called “class III” polypeptides are located within this multigene region [47–49]. About 40% of the expressed MHC genes encode proteins related to immune defense [48]. Whereas the classical class I and class II transplantation antigens are expressed on cells and tissues (with the exception of proteins involved in antigen processing and presentation of antigens to the immune system, such as LMPs, TAPs, and Tapasin), the class III antigens are secreted proteins which do not play a role in tissue acceptance or graft rejection. Class III antigens comprise proteins with immune functions such as components of the complement cascade (C2, C4, and factor B), cytokines (TNF-*α*, LTA, LTB), steroid metabolism (Cyp21B), heat shock proteins (hsp70), and many other genes not directly associated with immune responses [50]. For historical reasons, human MHC polypeptides are called “human leukocyte antigens” (HLA) and mouse MHC proteins “histocompatibility 2” (H-2) antigens.

In humans, the MHC is the most gene-dense region of the genome, and the MHC genes themselves are the most polymorphic genes known so far. Among the ~3 billion base pairs of the human or murine genome, arranged on 23 and 20 chromosomes, respectively, there are 20,000–30,000 protein-coding genes [51–53]. That means that an average of one gene was found for every 100,000 to 150,000 base pairs. The human MHC, however, contains more than 120 functional genes and additional nonfunctional pseudogenes in both mice and humans distributed over 3.6 Mbp [54–57]. The outcome of this is an average of approximately one gene for every 30,000 base pairs.

MHC molecules are codominant expressed and clustered in so-called “haplotypes”. The term was introduced by Ceppellini et al. (in 1967), who used familial genotype data, to explain the coinheritance of alleles at two closely linked loci [58]. This organization is thought to facilitate recombination events that generate new alleles and therefore, contribute to the high polymorphism of MHC proteins. Polymorphism derives from the creek word “ *πολυμορφία*” (polymorphia) and means “many or complex shapes”. The polymorphism found in the MHC class II genes is generally limited to exon 2, which encodes the peptide-binding groove [59]. Due to the high frequency of MHC alleles, most individuals will be heterozygous for each different MHC gene locus. Each MHC molecule in the population has a different spectrum of peptide binding. This insures that no one pathogen can destroy the whole population by developing protein sequences that are incapable of binding to an MHC molecule, and thus evading the immune system (Figure 3).

In contrast to humans the number of MHC (H-2) alleles is strongly reduced in inbred mice because of the homozygosity at their MHC loci. As many peptides are not recognized by the remaining alleles/haplotypes, these mice often have an impaired immune response against pathogens. In fact, the MHC genes of mice were first called “immune response (Ir) genes because of strain-dependent defects in responses to certain antigens [38].

### 2.3. Evolution of MHC Diversity

In the sixties and seventies, two different models have been developed to explain the high heterogeneity of the MHC genes: Negative frequency dependence (rare allele advantage) and heterozygote advantage (overdominance model) [60–62]. The negative frequency dependence postulates that rare MHC alleles (of recent origin) may have a selective advantage, as no pathogen may be adapted to it [63]. The overdominance model states that polymorphism will be advantageous because heterozygous individuals are able to recognize a wider range of pathogens and parasites [60]. A main difference between these two types of (balancing) selection is that overdominance is based upon a stable polymorphism, whereas a polymorphism maintained by frequency dependence will be dynamic [64]. However, there is still a controversy, if the heterozygote advantage on its own is sufficient to explain the high degree of MHC polymorphism [65]. For instance, it has recently been shown that balancing selection can also result from MHC-dependent choice of mates [66].

Evolution of MHC genes and alleles is driven by the need to maximize peptide binding diversity in order to recognize a maximum of potential pathogens. Polymorphism and polygeny are two (independent) genetic mechanisms for increasing variety of MHC class I and class II proteins. Polygeny acts on the individual level, whereas polymorphism is (primarily) a population-relevant criterion. Thus, a maximum number of class I and II genes would ensure the greatest conceivable protection of a single individual against pathogens. However, polygeny is limited by a mechanism called “MHC restriction”: T cells recognize fragmented antigens (self and foreign) only in conjunction with MHC proteins [42, 67]. To avoid autoimmune reactions, T cells that strongly react with MHC molecules presenting self-peptides are deleted. In consequence of these opposed requirements, the immune surveillance is a delicate balance between self and foreign as well as between (self-)tolerance and immune response. Furthermore, these two opposing demands create a dilemma: On the one hand, many MHC genes would present a maximum of different peptides but on the other hand, the presentation of many different self-antigens would strongly reduce the T cell diversity. Thus, MHC restriction limits T cell antigen recognition and response. As a consequence of this, the diversity of MHC class I and II proteins of a single individual is limited (and optimized) to six different molecules (3 genes × 2 alleles). The optimal number is called “immunogenetic optimum” [68]. Due to the limited number of MHC genes, some agents may evolve polypeptides that evade the immune system of single individuals, but the enormous polymorphism within a population diminishes the possibility that a pathogen can exterminate a whole species (individual C). However, there is a major drawback of this kind of defense strategy: if the size of a population decreases strongly, some MHC haplotypes will disappear, leading to a reduction of MHC diversity, which in turn will negatively affect survival of the population [69]. In summary, the number of different MHC genes is a delicate balance between the key requirement of an entire population/species and the core requests of its individuals.

## 3. How Is Lupus Erythematosus Influenced by the MHC?

Variations within the MHC locus seem to be associated with a great variety of autoimmune diseases. Consequently, the contribution of HLA genes to lupus pathology has recently been extensively studied [21, 70–72]. However, due to the extensive linkage disequilibrium among alleles throughout this locus, the causal relationship between these MHC variations and autoimmune pathogenesis have remained elusive for the great majority of these diseases, including lupus [73].

**Figure 1:**
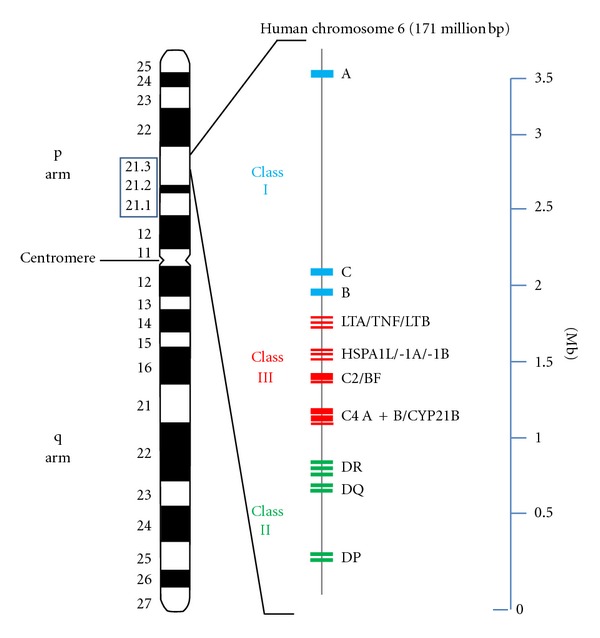
HLA gene cluster and lupus susceptibility genes on human chromosome 6. Ideogram of chromosome 6 (left) and schematic diagram of the MHC-complex-associated genes ranging from 6p21.1 to 6p21.3 (middle). The class I gene complex contains three major loci (A, C, and B), as well as additional (unmentioned) loci. The resulting class I polypeptides associate with the invariable beta-2 microglobulin, encoded by a gene on chromosome 15. The HLA-B locus is known as the most polymorphic gene within the human genome. Class II MHC molecules are composed of two glycosylated polypeptide subunits (called *α* and *β* chain) of approximately equal length. Whereas HLA-DP and -DQ code for one alpha- and one beta-chain polypeptide, respectively, the genetics of HLA-DR is more complex: It consists of one locus coding for the alpha subunit and 4 loci coding for beta subunits. Unlike the other DR loci, DRA is not polymorphic. Even though the DR *β*-chain is encoded by 4 loci, no more than two are present on a single chromosome. DRB1 is the most polymorphic gene of the class II locus. Class I and class II antigens are membrane proteins whereas almost all class III polypeptides are serum proteins (including the complement components C2, C4A, C4B, and factor B) or can be detected in other body fluids. Therefore, the term “class III” is misleading, as this locus does not contain a distinct class of genes. The coding regions of the genes are shown as small blue (class I), green (class II), and red (class II) rectangles, respectively. Abbreviations: LTA: lymphotoxin A, LTB: lymphotoxin B, TNF: tumor necrosis factor alpha, HSPA1L: heat shock 70 kDa protein 1-like, HSPA1A: heat shock 70 kDa protein 1A, HSPA1B: heat shock 70 kDa protein 1B, BF: complement factor B, CYP21B: cytochrome P450 21-hydroxylase and Mb: mega base pairs.

Although the pathogenesis of the disease is still poorly understood and a number of environmental factors have been postulated, genetic predisposition is clearly a major risk parameter for SLE [74, 75]. There is strong evidence for a genetic component based upon a high concordance rate of SLE in monozygotic twins as well as the occurrence of SLE in 5–12% of the relatives of affected patients [76–79]. The complex nature of SLE reflects a polygenic inheritance of the disease rather than a monogenic mode. Several genes are known to contribute to SLE susceptibility [80, 81], because they affect key pathways, implicating immune complexes, host immune signal transduction, and interferon pathways (reviewed in [82]). Only in a small proportion of patients (<5%), a single gene seems to be responsible for the disease onset. Many of these genes relate to the early complement components from which the C2 and C4 genes are linked to the MHC (Figure 1 and [83–85]).

The mechanisms underlying antigen recognition are of great importance to human autoimmune diseases. A number of genes have been claimed to be associated with susceptibility to anti-self responses. Because of their considerable heterogeneity, the immunoglobulin genes, the T-cell receptor genes, and the major histocompatibility complex (MHC) genes have soon been suspected of playing a distinct role in the pathology of lupus and other autoimmune diseases. Particularly, the MHC class II allotypes HLA-DR2 and -DR3 seem to be related to (and/or positively correlated with) lupus disease [86–88]. Genes, like angiotensin-converting enzyme (ACE) or angiotensinogen (AGT), that specifically increase kidney susceptibility to lupus pathogenesis have also been described [89].

Advances in high throughput technology have enabled the genotyping of hundreds of thousands of single nucleotide polymorphisms (SNPs) in a single individual and genome-wide association studies (GWAS) in lupus patients [90]. GWAS in European- or East Asian-ancestry populations [91–94] and high-density screenings [20, 95] have identified several independent SNPs in the MHC region associated with SLE. Some of these SNPs could be confirmed in a recent targeted association study [96]. GWAS may also been used to decipher complex ethnic disparities in SLE prevalence rates. For unknown reasons, the prevalence of lupus in African and Hispanic Americans is two to fivefold higher compared to Americans of European ancestry [97]. A recent SNP screening of the MHC region revealed for independent SNP signals for African American women [98]. The strongest signal of this study (the SNP rs9271366), was also associated with SLE in a previous Chinese GWA study of Han and coworkers [91]. It has also been shown by GWAS that several established non-MHC lupus loci are not related to other autoimmune diseases, which suggests a limited genetic overlap between these diseases and SLE [99]. In summary, it can be stated that genome-wide and -targeted association studies, despite of their methodological and application-related limitations, are useful tools to localize lupus-associated genes.

In the past few years, progress has been made in identifying lupus susceptibility genes in mice [100, 101]. Meanwhile, a large number of lupus susceptibility loci have been detected in mouse models, and some of the corresponding susceptibility genes have been identified by now (reviewed in [10, 102–106]) including those linked to MHC [14, 107, 108]. An important milestone in murine lupus genetics was the identification of the SLE loci 1–3 by Mohan et al. and Morel et al. in NZM2410 mice [109–111], a lupus-prone strain derived from a cross between NZB and NZW mice [112]. The identification of these loci provided the starting basis for a rapidly growing number of publications that dissected the role of single loci or genes in lupus development [113–119]. Several B6-based lupus congenic strains has been characterized, that carry the NZM2410-derived SLE-susceptibility loci *Sle1*, *Sle2*, and *Sle3* (reviewed in [17]). It has been shown that these three loci act in an additive way and that the coexpression of them is necessary to develop the full severity of the disease [107, 120]. Subsequently, it has been demonstrated by congenic dissection and polygenic analyses that both protective suppressor and harmful susceptibility loci form the genetic basis for murine lupus and that they act in a highly complex manner that involves several genes [121, 122]. Meanwhile, for a subset of these murine genes, involvement in human SLE has been established [17].

Based upon these models, there is considerable evidence that single MHC genes contribute to the development of systemic lupus erythematosus [26, 123–125]. However, in both mice and humans, lupus susceptibility results from accumulating effects of a large number of individual gene variants [126] of which the MHC-linked loci are reviewed below.

### 3.1. MHC Class I Genes

The association between MHC loci and susceptibility to lupus has been known since 1971, when HLA-B8 was shown to be associated with this disease [21]. In particular, the ancestral haplotype A1-B8-DR3 has been linked to lupus susceptibility [127–130]. Nevertheless, early studies have focused upon MHC class II genes in lupus pathogenesis, since class II-restricted CD4^+^ T cells have been associated with the generation of autoantibodies [131]. Although the dysregulation of class I levels is predicted to result in autoimmunity [132], the relevance of MHC class I proteins to lupus, however, is less clear. Recent studies have implicated a distinct role for MHC (H-2) class I molecules in mouse lupus pathogenesis: McPhee et al. could demonstrate that *β*2-microglobulin-deficient (*β*2m) BXSB-Yaa and -SJL mice (i.e., mice deficient in class I antigen presentation) developed much more aggressive and lethal forms of a lupus-like disease that characterizes these strains [133]. These results are in line with previous findings in the (NZB × NZW) F1 mouse model of lupus disease [125]. A more sophisticated role for class I proteins could be demonstrated for *β*2m-deficient MRL*/lpr* mice: While inhibiting nephritis, *β*2m deficiency accelerates spontaneous lupus skin disease [134]. In another report, Mozes et al. could show that MHC class I-deficient mice are resistant to experimental SLE, although these mice were not generally poor responders to antigen [135]. Furthermore, MHC class I-deficient MRL*/lpr* mice demonstrate a substantial reduction in CD4/CD8 double-negative (DN) T cells and symptoms of the lupus-like disease [136]. In summary, these results indicate that class I-dependent T cells are key players for the murine lupus-like syndrome.

### 3.2. MHC Class II Genes

SLE is associated with class II genes of the MHC, but it is not yet clear which haplotypes, genes, or alleles are primarily responsible for disease association. Initial reports looking at the involvement of HLA in SLE assumed a direct involvement of haplotypes containing DR2 and/or DR3 to disease pathogenesis [22, 137–140], but later reports indicated for both humans and mouse models that HLA DR molecules may have an increased association with the production and specificity of autoantibodies rather than with the disease itself [75, 141–143]. Meanwhile, an immense number of studies based on different ethnicities have identified HLA class II associations with SLE.

The presence of antinuclear antibodies (ANA) is a serological hallmark of lupus erythematosus (found in the serum of most patients) [144], and the role of HLA genes in autoantibody expression has been intensely researched, because it indicates the activation of autoaggressive B-cells and the breakdown of tolerance to self-antigens. A subspecies of antinuclear autoantibodies, called Ro/SSA (a ribonuclear protein) is present in 25–50% of SLE cases [145, 146] and the level and occurrence of theses autoantibodies correlate with the presence of HLA-DR2/DR3 and HLA-DQw1/DQw2 heterozygotes [147]. In mouse models, heterozygosity at the MHC (H-2) locus has also been associated with lupus susceptibility and enhanced autoantibody production [148, 149]. For (NZB × NZW) F1 hybrid mice, it has been hypothesized that H-2A or H-2E MHC class II genes are two likely candidates [81]. DQA1*0102 and DQA1*0301 alleles were observed to be strongly associated with the presence anti-Ro/La and anti-dsDNA antibodies in Chinese but not in a Malaysian control group [150]. However, a German lupus study showed that all HLA-DR and -DQ (homozygous and heterozygous) combinations appear with frequencies expected from the observed gene frequencies, suggesting that gene complementation at MHC class II loci seems not to contribute to lupus susceptibility [151].

Other autoantibodies are detected in patients with SLE but the HLA associations with these are less clear. Antiphospholipid antibodies are frequently observed in patients with SLE [152–154] and a significant association of DR7-positive patients (in linkage disequilibrium with the HLA-DR gene B4) that carry anticardiolipin antibodies could be observed by Savi et al. [155]. Azizah et al. found a significant association of the DQB1*0601 allele with anti-Sm/RNP, DR2 with anti-Ro/La, and DR2, DRB1*0501, and DRB1*0601 with anti-dsDNA antibody expression [156].

It has been shown that the HLA haplotype DR3-DQ2-C4AQ0 is strongly associated with SLE in Caucasians [157, 158]. A strong association with lupus was also determined by DNA typing for DQA1* 0501 in Scandinavian patients [159]. However, this allele was in linkage disequilibrium with DR3 and DR5. A strong association to SLE is found with DRB1*03 and DOB1*0201 alleles of central European patients [160]. A genetic predisposition of HLA DR2- and/or HLA DR3-containing haplotypes for SLE has also been described for German, Kuwaiti, and Chinese lupus patients [161–163].

Strong associations of class II genes with lupus susceptibility have also been shown by GWA studies. Studies based on sequence length polymorphisms in European populations identified a potential association of the class II HLA-DRB1 alleles HLA-DRB1*08:01, -*03:01, and -*15:01 with SLE [73, 164]. Two of these alleles (HLA-DRB1*03:01 and -*15:01) have also been identified in a recent study of the IMAGEN consortium using high-density SNP typing across the MHC [20]. In a study of Ruiz-Narvaez et al. the strongest SLE-associated SNP was the rs9271366 near the HLA-DRB1 gene [98]. This SNP was also associated with higher risk of SLE in a previous GWAS [91]. Although there are hardly any GWAS results concerning class III genes, the SNP rs419788 in intron 6 of the class III gene SKIV2L was found to be independently associated with SLE [165]. However, in a recent report this SNP was not found to be independent from the rs3135391 (HLA-DRB1*15:01) signal [96].

In summary, these results indicate that both DR2 and DR3 and their associated DQ alleles seem to play a role in SLE [146, 166]. However, most of the results concerning the contribution of individual MHC class II polymorphisms to SLE have been obtained from population-based case-control studies and need to be confirmed in family-based studies [146].

MRL/*lpr* mice spontaneously develop aggressive autoimmune kidney disease characterized by an immune complex glomerulonephritis, which is associated with increased (or de novo) renal expression of major histocompatibility complex (MHC) class II molecules and a massive systemic expansion of CD4-CD-double negative (DN) T cells [167–169]. However, these mice are homozygous for the H-2^*k*^ haplotype, which is shared by several other strains, that do not develop lupus-like symptoms. In addition, it has been shown that genes encoded within or closely linked to the MHC region regulate autoantigen selection and isotype switching to IgG3 but have minimal effect on end-organ damage or survival in MRL/*lpr * mice [170]. On the other hand, MHC (H-2) class II expression appears to be required for the development of autoaggressive CD4^+^ T cells involved in autoimmune nephritis, because MHC class II-deficient MRL*/lpr* mice do neither produce serum anti-DNA antibodies nor develop proliferative renal disease in contrast to their wild-type counterparts [168].

In contrast to New Zealand black (NZB) and New Zealand white (NZW) mice, F1 hybrids of these strains (with a H-2^*d*/*z*^ haplotype) spontaneously develop a severe lupus-like immune complex glomerulonephritis associated with the production of antinuclear autoantibodies [171]. Morel et al. have focused on the genetic dissection of lupus-prone NZM2410 mice, which are derived from this cross [110, 112] and identified four epistatic modifiers (*Sles1–4*) by linkage analysis. The cumulative effect of these suppressive loci accounts for the benign autoimmunity in NZW mice [122]. The strongest one, *Sles1*, being encoded by an MHC (H-2^*z*^) class II locus, was sufficient to completely prevent autoimmunity initiated by Sle1 in (NZW × B6.NZMc1) F1 mice.

MHC H-2^*d*/*z*^ heterozygosity (H-2^*d*^ of NZB and H-2^*z*^ of NZW mice) promotes lupus disease, as congenic H-2^*d*/*d*^and H-2^*z*/*z*^ homozygous crosses do not develop severe disease [172, 173]. On the other hand, Zhang and coworkers found that H-2A^*d*/*d*^ homozygous (NZB × NZW) F1 mice lacking H-2E molecules developed severe SLE similar to that seen in wild-type F1 mice, whereby the effect of H-2E is greatly influenced by the haplotype of H-2A molecules [174]. The authors propose two different mechanisms to explain their results: First, compared with H-2^*d*/*d*^ F1 mice, the self-antigen presenting capacity of DCs in H-2^*d*/*z*^ F1 is much higher, so that effects of E molecules may be insufficient for disease suppression and, alternatively, generation of H-2^*d*/*z*^ F1 unique self-reactive T cells restricted to haplotype mismatched H-2A*α*/*β* heterodimers in the thymus may play a role in an H-2E molecule-independent manner. However, one should keep in mind that H-2^*d*/*z*^ heterozygosity is a necessary but not sufficient condition for the development of autoimmunity in NZB/W F1 mice [175]. Kotzin and coworkers wanted to dissect the role of *Ea* ^z^, *Eb* ^z^, *Aa* ^z^, and *Ab* ^z^ MHC class II molecules to lupus susceptibility, but they could not observe an increased contribution of these polypeptides to the seriousness of the disease in transgenic approaches [26, 123].

BXSB mice spontaneously develop a male-biased lupus-like syndrome that is accelerated by the *Yaa* (Y-linked autoimmune accelerator) gene [9, 176]. The BXSB MHC locus (H-2b haplotype) plays a crucial role in disease expression since congenic BSXB.H-2d mice have a less severe syndrome [2]. As B6*·*Yaa (H-2b/b) mice do not develop lupus symptoms, there are also non-MHC-linked genes in the BSXB genome that contribute to disease development [104]. It has been shown that lupus was initiated by a translocation of 17 genes, including *TLR7*, from the X to the Y chromosome [3, 4]. TLR7 overexpressing transgenic mice have demonstrated that duplication of the *TLR7 *gene is the sole requirement for this accelerated autoimmunity, as reduction of TLR7 gene dosage abolishes the Yaa phenotype [177]. Furthermore, TLR7 and additional nucleic acid-binding TLRs, consisting of the toll-like receptors 3 and 9, exacerbate lupus-like disease in other autoimmune-prone strains [178]. Although a TLR7 gene copy-number variation could be detected in the human genome, it was not significantly increased among SLE patients as compared with the healthy control group, and no significant concordance between the number of gene copies and the SLE phenotype was found [179]. However, other reports describe SNPs in the human *TLR7* gene that associate with lupus [180, 181]. García-Ortiz and coworkers reported an association between increased *TLR7* gene copy numbers and childhood-onset SLE in the Mexican children [182].

However, even after more than 30 years of research, the precise contribution of HLA class II genes to lupus pathogenesis remains ambiguous and is still a matter of discussion.

### 3.3. MHC Class III Genes

**Figure 2:**
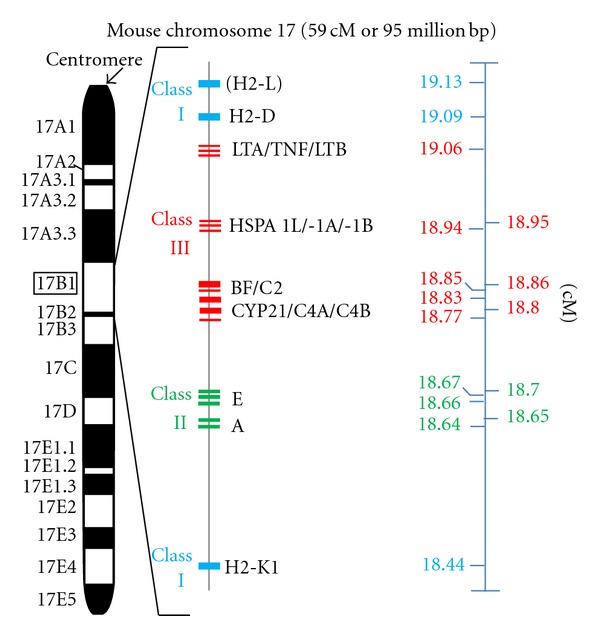
H-2 gene cluster and lupus susceptibility genes on mouse chromosome 17. Ideogram of chromosome 17 (middle) and schematic diagram of the MHC-complex-associated genes at 17 B1 (boxed left). The coding region of the genes is shown as small blue (class I), green (class II), and red (class II) rectangles, respectively. There are 11 mouse MHC subclasses, ranging from 18.4 to 20.3 centimorgan (cM). However, the organization of the “classical” MHC classes is similar in human and mouse. The MHC class I consists of two major loci, K and D, which are (unlike the human MHC class I counterpart) separated by the class II and class III genes. The class II gene complex is known as the I region (from “immune response”) and the class II genes are also termed “Ir genes”. Class III lupus susceptibility genes are tumor necrosis factor alpha (TNF), cytochrome P450 21-hydroxylase a1 (CYP21) as well as the complement factors C2, C4A, and C4B, respectively. Note, that cM is a nonlinear genetic distance unit of recombinant frequency, which is influenced by several factors. Further abbreviations: LTA: lymphotoxin A, LTB: lymphotoxin B, HSPA1L: heat shock 70 kDa protein 1-like, HSPA1A: heat shock 70 kDa protein 1A, HSPA1B: heat shock 70 kDa protein 1B, and BF: complement factor B. Source of mapping data: IMGT information system (http://www.imgt.org/IMGTrepertoireMHC/LocusGenes/): Artzt et al. (1991), Mammalian Genome 1: 280ff. [232], and Endo et al. [233], Gene 205: 19ff. [233].

**Figure 3:**
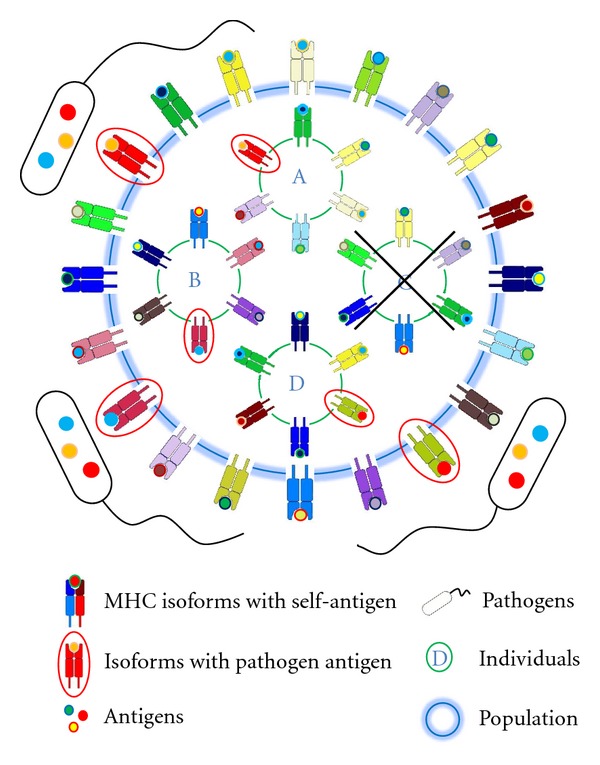
Protective effect of MHC polymorphism on populations (simplified scheme). Evolution of MHC genes and alleles is driven by the need to maximize peptide binding diversity in order to recognize a maximum of potential pathogens. Thus, the extreme polymorphism of MHC molecules of vertebrates is thought to reflect a pathogen-driven selection. This insures that no germ can exterminate the whole population by developing peptides that cannot be bound by any MHC molecule. However, compared to the enormous diversity of MHC molecules within a population (outer circle), their heterogeneity within a single individual is restricted to a few different MHC polypeptides (individuals A–D). This can be attributed to a mechanism called “MHC restriction” (see MHC chapter) that limits polygeny of the MHC genes (in general to 3 genes per MHC class I and class II). As a consequence some agents may evolve polypeptides that evade the immune system of single individuals (individual C) and harm or even kill them. In consequence of these opposed requirements, the immune surveillance is a delicate balance between (self-)tolerance and immune response that ensures survival of a population and/or a species at the expense of single individuals.

Class III genes of the MHC encode proteins that are not involved in antigen presentation (Figures 1 and 2). C2, C4A, C4B, and factor B are complement components that constitute both the C3 convertases of the classical and alternative pathway [183, 184]. Tumor necrosis factor alpha (TNF-*α*) and its related proteins lymphotoxin-*α* and -*β* are immune modulating cytokines of the TNF superfamily [185, 186], and the heat shock protein 70 (Hsp70) orthologues are a triplet of genes, which are important components of the chaperone machinery [187, 188].

#### 3.3.1. Complement Components

The complement system plays an important role in innate and adaptive immunity [189]. Its main biological function is to recognize foreign particles, macromolecules, and apoptotic cells, and to support their elimination either by opsonisation or lysis [190]. Although rare, inappropriate complement activation as well as complement deficiencies are involved in the pathophysiology of systemic lupus erythematosus [25, 191, 192].

Lupus is casually associated with the homozygous deficiency of the most early components of the complement activation pathway (C1q, C1r, C1s) [189, 193]. However, MHC-linked C2 and C4 deficiencies are also associated with SLE [194], and approximately 40% of C2 deficient individuals develop SLE-like symptoms [195]. In fact, homozygous C2 deficiency is thought to be the most common inherited complement defect associated with lupus [196, 197]. In addition, Fielder et al. found a high frequency of null alleles at the C2, C4A, and C4B loci in families of SLE patients [198]. In humans and mice, C4 is encoded by two tandemly arranged genes (C4A and B) within the MHC ([199] and Figures 1 and 2). About 40 protein variants for C4 have been documented [200]. It has been shown that low copy numbers of the C4 gene are a risk factor for SLE in European Americans [201] and a large C4A-CYP21A gene deletion (particularly associated with HLA-B44, -DR2, and -DR3 alleles) in black Americans [202]. On the other hand, C3 deficiency is only rarely associated with lupus development, because homozygous hereditary C3 deficiency is a seldom genetic disease [203]. It is thought that absence of complement proteins results in a defective immune complex clearance and, in consequence, to a deposition of the complexes in various organs [204, 205]. An alternative hypothesis postulates that self-reactive B cells, which are specific for lupus autoantigens, are not effectively silenced (or eliminated) without complement [206]. In fact, recent findings suggest, that enhanced B cell function is the defining pathogenic event of lupus pathogenesis, leading to autoimmunity and organ damage [207].

Aberrant splicing of the C4 mRNA (caused by an intronic insertion of the B2 sequence in the C4 gene) is the basis for low C4 expression in H-2^*k*^ mice, such as lupus-prone MRL mice [208, 209]. An association between complement deficiency and SLE has also been shown for complement-deficient mouse models [210]. C1q- and C4-deficient mice develop a lupus-like disease and exhibit impaired clearance of apoptotic cells [211]. Indeed, apoptotic cells are thought to be a major source of the autoantigens of SLE [212]. This has led to the hypothesis that the delayed clearance of apoptotic material leads to a persistence of proinflammatory activities which may then initiate autoimmunity.

#### 3.3.2. Heat Shock Protein (HSP) Genes

Heat shock proteins (hsp) are highly conserved proteins that regulate protein folding. They are induced by a variety of stresses like heat, growth factors, inflammation, and infection [213]. The expression of hsp90 is found to be increased in the mononuclear cells of about one-fourth of SLE patients and antibodies to this protein are detected in patients with SLE [146]. Levels of hsp90 protein in SLE patients seem to correlate with IL-6 and hsp90 autoantibody levels, supporting the following scenario: Elevated levels of IL6 in SLE patients induce higher levels of hsp90 protein which in turn results in the production of hsp90 autoantibodies [214].

Another heat shock protein that play a role in SLE pathogenesis is HSPA1B, a member of the hsp70 gene family. The HSPA1A, HSPA1B, and HSPA1L are MHC class III genes in murines and humans, which code for highly homologous polypeptides [215]. HSPA1B encodes a polypeptide that is thought to be involved in disease susceptibility [216]. Association of a polymorphism (A to G transition) in the coding region of the HSPA1B gene with SLE in African Americans has been reported in a case-control study [217].

#### 3.3.3. Tumour Necrosis Factor (TNF) Gene

Tumour necrosis factor alpha (TNF-*α*) is an inducible member of the TNF/TNFR superfamily with a broad range of immunological effects [218]. Macrophages are the major source of TNF-*α*, although it can be produced by many other cell types as well [219]. It is generally known as a proinflammatory cytokine, stimulating the acute phase response and increasing MHC class I and II expression as well as antigen-driven lymphocyte proliferation [220–222]. Dysregulation of TNF-*α* production has been implicated in a variety of human diseases, including lupus. A rare polymorphism (G to A transition) in the promoter region has been found to be increased in patients with SLE in a case-control study [223, 224], which is probably due to linkage disequilibrium with DR3 [225]. However, other reports based on Caucasian SLE patients describe an independent contribution of TNF polymorphisms and HLA-DR3 to SLE susceptibility [226, 227].

As in humans, the murine TNF-*α* gene is located within the MHC [228]. The NZW mouse strain carries a unique TNF allele, that expresses only limited amounts of TNF-*α* [229]. It has been proposed that this polymorphism ameliorates murine lupus symptoms [228, 230] and, indeed, it has been shown by Kontoyiannis and Kollias, that autoimmunity and lupus nephritis is accelerated in NZB mice with an engineered heterozygous deficiency in tumor necrosis factor [231].

## 4. Concluding Remark

The MHC genes including TNF*α*, HSP70, and class II genes have been associated with systemic lupus erythematosus. However, in most cases, genetic susceptibility to lupus is not caused by a single gene or allelic variation. Defects in complement genes are well-documented exceptions, which may predispose to lupus because of the persistence of antibody complexes or activation of self-reactive B cells. The role of TNF*α*, HSP70, or MHC class II gene loci in lupus pathology is more difficult to evaluate. This is due, among others, to the linkage disequilibrium of the MHC, which makes it difficult to prove a direct contribution of single genes or alleles to lupus susceptibility. Furthermore, the identification of susceptibility or suppressor genes is complicated by the plain fact that SLE is a highly heterogeneous disease that appears when susceptibility and suppressor loci are unbalanced. In addition, environmental, epigenetic, hormonal, and infectious factors may alter the epigenetic status quo and may trigger lupus in genetically-susceptible individuals. On the other hand, analysing the influence of environmental factors on the epigenetic status of well-defined MHC haplotypes or MHC gene polymorphisms may open promising perspectives for future studies.

For these reasons, deciphering the contribution of MHC locus and its gene products to the pathogenesis of human and murine lupus will add the next important piece of the puzzle that will further clarify the etiology of this complex autoimmune disease.

## References

[1] Rahman A, Isenberg DA (2008). Systemic lupus erythematosus. *The New England Journal of Medicine*.

[2] Merino R, Fossati L, Lacour M, Lemoine R, Higaki M, Izui S (1992). H-2-1inked control of the Yaa gene-induced acceleration of lupus-like autoimmune disease in BXSB mice. *European Journal of Immunology*.

[3] Subramanian S, Tus K, Li QZ (2006). A Tlr7 translocation accelerates systemic autoimmunity in murine lupus. *Proceedings of the National Academy of Sciences of the United States of America*.

[4] Pisitkun P, Deane JA, Difilippantonio MJ, Tarasenko T, Satterthwaite AB, Bolland S (2006). Autoreactive B cell responses to RNA-related antigens due to TLR7 gene duplication. *Science*.

[5] Chu JL, Drappa J, Parnassa A, Elkon KB (1993). The defect in Fas mRNA expression in MRL/lpr mice is associated with insertion of the retrotransposon, ETn. *The Journal of Experimental Medicine*.

[6] Watanabe-Fukunaga R, Brannan CI, Copeland NG, Jenkins NA, Nagata S (1992). Lymphoproliferation disorder in mice explained by defects in Fas antigen that mediates apoptosis. *Nature*.

[7] Lynch DH, Watson ML, Alderson MR (1994). The mouse fas-ligand gene is mutated in gld mice and is part of a TNF family gene cluster. *Immunity*.

[8] Takahashi T, Tanaka M, Brannan CI (1994). Generalized lymphoproliferative disease in mice, caused by a point mutation in the Fas ligand. *Cell*.

[9] Andrews B, Eisenberg RA, Theofilopoulos AN (1978). Spontaneous murine lupus-like syndromes. Clinical and immunopathological manifestations in several strains. *The Journal of Experimental Medicine*.

[10] Theofilopoulos AN, Dixon FJ (1985). Murine models of systemic lupus erythematosus. *Advances in Immunology*.

[11] Cohen PL, Eisenberg RA (1991). Lpr and gld: single gene models of systemic autoimmunity and lymphoproliferative disease. *Annual Review of Immunology*.

[12] Izui S, Iwamoto M, Fossati L, Merino R, Takahashi S, Ibnou-Zekri N (1995). The Yaa gene model of systemic lupus erythematosus. *Immunological Reviews*.

[13] Nguyen C, Limaye N, Wakeland EK (2002). Susceptibility genes in the pathogenesis of murine lupus. *Arthritis Research*.

[14] Vyse TJ, Kotzin BL (1996). Genetic basis of systemic lupus erythematosus. *Current Opinion in Immunology*.

[15] Santiago-Raber ML, Laporte C, Reininger L, Izui S (2004). Genetic basis of murine lupus. *Autoimmunity Reviews*.

[16] Li L, Mohan C (2007). Genetic basis of murine lupus nephritis. *Seminars in Nephrology*.

[17] Morel L (2010). Genetics of SLE: evidence from mouse models. *Nature Reviews Rheumatology*.

[18] Ballestar E, Esteller M, Richardson BC (2006). The epigenetic face of systemic lupus erythematosus. *Journal of Immunology*.

[19] Javierre BM, Richardson B (2011). A new epigenetic challenge: systemic lupus erythematosus. *Advances in Experimental Medicine and Biology*.

[20] Rioux JD, Goyette P, Vyse TJ (2009). Mapping of multiple susceptibility variants within the MHC region for 7 immune-mediated diseases. *Proceedings of the National Academy of Sciences of the United States of America*.

[21] Grumet FC, Coukell A, Bodmer JG, Bodmer WF, McDevitt HO (1971). Histocompatibility (HL-A) antigens associated with systemic lupus erythematosus. A possible genetic predisposition to disease. *The New England Journal of Medicine*.

[22] Harley JB, Moser KL, Gaffney PM, Behrens TW (1998). The genetics of human systemic lupus erythematosus. *Current Opinion in Immunology*.

[23] Criswell LA (2008). The genetic contribution to systemic lupus erythematosus. *Bulletin of the NYU Hospital for Joint Diseases*.

[24] Tan FK, Arnett FC (1998). The genetics of lupus. *Current Opinion in Rheumatology*.

[25] Walport MJ (2002). Complement and systemic lupus erythematosus. *Arthritis Research*.

[26] Rozzo SJ, Vyse TJ, David CS, Palmer E, Izui S, Kotzin BL (1999). Analysis of MHC class II genes in the susceptibility to lupus in New Zealand mice. *Journal of Immunology*.

[27] Gorer PA (1937). The genetic and antigenic basis of tumour transplantation. *Journal of Pathology and Bacteriology*.

[28] Medawar PB (1961). Peter Alfred Gorer. 1907–1961. *Biographical Memoirs of Fellows of the Royal Society*.

[29] Medawar PB (1944). The behaviour and fate of skin autografts and skin homografts in rabbits: a report to the War Wounds Committee of the Medical Research Council. *Journal of Anatomy*.

[30] Starzl TE (1995). Peter Brian Medawar: father of transplantation. *Journal of the American College of Surgeons*.

[31] Snell GD (1948). Methods for the study of histocompatibility genes. *Journal of Genetics*.

[32] Snell GD, Higgins GF (1951). Alleles at the histocompatibility-2 locus in the mouse as determined by tumor transplantation. *Genetics*.

[33] Klein J (2001). George Snell’s first foray into the unexplored territory of the major histocompatibility complex. *Genetics*.

[34] Dausset J (1958). Iso-leuko-antibodies. *Acta Haematologica*.

[35] Charron DJ (2009). In memoriam: Jean Dausset (1916–2009). *Tissue Antigens*.

[36] Payne R, Tripp M, Weigle J, Bodmer W, Bodmer J (1964). A new leukocyte isoantigen system in man. *Cold Spring Harbor Symposia on Quantitative Biology*.

[37] Levine BB, Ojeda A, Benacerraf B (1963). Studies on artificial antigens. Iii. the genetic control of the immune response to hapten-poly-L-lysine conjugates in guinea pigs. *The Journal of Experimental Medicine*.

[38] Benacerraf B, McDevitt HO (1972). Histocompatibility-linked immune response genes. *Science*.

[39] Ellman L, Green I, Martin WJ, Benacerraf B (1970). Linkage between the poly-L-lysine gene and the locus controlling the major histocompatibility antigens in strain 2 guinea pigs. *Proceedings of the National Academy of Sciences of the United States of America*.

[40] McDevitt HO, Tyan ML (1968). Genetic control of the antibody response in inbred mice. Transfer of response by spleen cells and linkage to the major histocompatibility (H-2) locus. *The Journal of Experimental Medicine*.

[41] Mcdevitt HO, Chinitz A (1969). Genetic control of the antibody response: relationship between immune response and histocompatibility (H-2) type. *Science*.

[42] Zinkernagel RM, Doherty PC (1974). Restriction of in vitro T cell mediated cytotoxicity in lymphocytic choriomeningitis within a syngeneic or semiallogeneic system. *Nature*.

[43] Brewerton DA, Hart FD, Nicholls A, Caffrey M, James DC, Sturrock RD (1973). Ankylosing spondylitis and HL-A 27. *The Lancet*.

[44] Schlosstein L, Terasaki PI, Bluestone R, Pearson CM (1973). High association of an HL-A antigen, W27, with ankylosing spondylitis. *The New England Journal of Medicine*.

[45] Klein J, Figueroa F, Klein D (1982). H-2 haplotypes, genes, and antigens: second listing. I. Non-H-2 loci on chromosome 17. *Immunogenetics*.

[46] Figueroa F, Tewarson S, Neufeld E, Klein J (1982). H-2 haplotypes of strains DBR7, B10.NZW, NFS, BQ2, STU, TO1, and TO2. *Immunogenetics*.

[47] Beck S, Geraghty D, Inoko H, Rowen L (1999). Complete sequence and gene map of a human major histocompatibility complex. *Nature*.

[48] Stewart CA, Horton R, Allock RJN (2004). Complete MHC haplotype sequencing for common disease gene mapping. *Genome Research*.

[49] Horton R, Wilming L, Rand V (2004). Gene map of the extended human MHC. *Nature Reviews Genetics*.

[50] Dressel R, Walter L, Günther E (2001). Genomic and functional aspects of the rat MHC, the RT1 complex. *Immunological Reviews*.

[51] Church DM, Goodstadt L, Hillier LW (2009). Lineage-specific biology revealed by a finished genome assembly of the mouse. *PLoS Biology*.

[52] Lander ES, Linton LM, Birren B (2001). Initial sequencing and analysis of the human genome. *Nature*.

[53] Waterston RH, Lindblad-Toh K, Birney E (2002). Initial sequencing and comparative analysis of the mouse genome. *Nature*.

[54] Trowsdale J (1995). ’Both man and bird and beast’: comparative organization of MHC genes. *Immunogenetics*.

[55] Geraghty DE, Koller BH, Pei J, Hansen JA (1992). Examination of four HLA class I pseudogenes: common events in the evolution of HLA genes and pseudogenes. *Journal of Immunology*.

[56] Geraghty DE, Koller BH, Hansen JA, Orr HT (1992). The HLA class I gene family includes at least six genes and twelve pseudogenes and gene fragments. *Journal of Immunology*.

[57] Beck S, Trowsdale J (2000). The human major histocompatibility complex: lessons from the DNA sequence. *Annual Review of Genomics and Human Genetics*.

[58] Ceppellini R, Curtoni ES, Mattiuz RL (1967). Genetics of leukocyte antigens: a family study of segregation and linkage. *Histocompatibility Testing*.

[59] Xu TJ, Sun YN, Wang RX (2011). Allelic polymorphism, gene duplication and balancing selection of the MHC class II DAB gene of Cynoglossus semilaevis (Cynoglossidae). *Genetics and Molecular Research*.

[60] Doherty PC, Zinkernagel RM (1975). Enhanced immunological surveillance in mice heterozygous at the H-2 gene complex. *Nature*.

[61] Clarke B, Kirby DRS (1966). Maintenance of histocompatibility polymorphisms. *Nature*.

[62] Bodmer WF (1972). Evolutionary significance of the HL-A system. *Nature*.

[63] Takahata N, Nei M (1990). Allelic genealogy under overdominant and frequency-dependent selection and polymorphism of major histocompatibility complex loci. *Genetics*.

[64] Slade RW, McCallum HI (1992). Overdominant vs. frequency-dependent selection at MHC loci. *Genetics*.

[65] De Boer RJ, Borghans JAM, Van Boven M, Keşmir C, Weissing FJ (2004). Heterozygote advantage fails to explain the high degree of polymorphism of the MHC. *Immunogenetics*.

[66] Apanius V, Penn D, Slev PR, Ruff LR, Potts WK (1997). The nature of selection on the major histocompatibility complex. *Critical Reviews in Immunology*.

[67] Zinkernagel RM, Doherty PC (1974). Immunological surveillance against altered self components by sensitised T lymphocytes in lymphocytic choriomeningitis. *Nature*.

[68] Wegner KM, Kalbe M, Kurtz J, Reusch TBH, Milinski M (2003). Parasite selection for immunogenetic optimality. *Science*.

[69] Sommer S (2005). The importance of immune gene variability (MHC) in evolutionary ecology and conservation. *Frontiers in Zoology*.

[70] Reinertsen JL, Klippel JH, Johnson AH (1978). B-lymphocyte alloantigens associated with systemic lupus erythematosus. *The New England Journal of Medicine*.

[71] Gibofsky A, Winchester RJ, Patarroyo M (1978). Disease associations of the Ia like human alloantigens. Contrasting patterns in rheumatoid arthritis and systemic lupus erythematosus. *The Journal of Experimental Medicine*.

[72] Scherak O, Smolen JS, Mayr WR (1980). HLA-DRw3 and systemic lupus erythematosus. *Arthritis and Rheumatism*.

[73] Fernando MMA, Stevens CR, Walsh EC (2008). Defining the role of the MHC in autoimmunity: a review and pooled analysis. *PLoS Genetics*.

[74] Michel M, Johanet C, Meyer O (2001). Familial lupus erythematosus: clinical and immunologic features of 125 multiplex families. *Medicine*.

[75] Kaplan D (1984). The onset of disease in twins and siblings with systemic lupus erythematosus. *Journal of Rheumatology*.

[76] Arnett FC, Reveille JD, Wilson RW (1984). Systemic lupus erythematosus: current state of the genetic hypothesis. *Seminars in Arthritis and Rheumatism*.

[77] Block SR, Winfield JB, Lockshin MD (1975). Studies of twins with systemic lupus erythematosus. A review of the literature and presentation of 12 additional sets. *American Journal of Medicine*.

[78] Deapen D, Escalante A, Weinrib L (1992). A revised estimate of twin concordance in systemic lupus erythematosus. *Arthritis and Rheumatism*.

[79] Giles I, Isenberg D (2001). Lupus in the family—analysis of a cohort followed from 1978 to 1999. *Lupus*.

[80] Sestak AL, Fürnrohr BG, Harley JB, Merrill JT, Namjou B (2011). The genetics of systemic lupus erythematosus and implications for targeted therapy. *Annals of the Rheumatic Diseases*.

[81] Vyse TJ, Kotzin BL (1998). Genetic susceptibility to systemic lupus erythematosus. *Annual Review of Immunology*.

[82] Moser KL, Kelly JA, Lessard CJ, Harley JB (2009). Recent insights into the genetic basis of systemic lupus erythematosus. *Genes and Immunity*.

[83] Agnello V (1978). Complement deficiency states. *Medicine*.

[84] Agnello V (1978). Association of systemic lupus erythematosus and SLE syndromes with hereditary and acquired complement deficiency states. *Arthritis and Rheumatism*.

[85] Sullivan KE, Wisnieski JJ, Winkelstein JA (1996). Serum complement determinations in patients with quiescent systemic lupus erythematosus. *Journal of Rheumatology*.

[86] Smolen JS, Klippel JH, Penner E (1987). HLA-DR antigens in systemic lupus erythematosus: association with specificity of autoantibody responses to nuclear antigens. *Annals of the Rheumatic Diseases*.

[87] Tjernström F, Hellmer G, Nived O, Truedsson L, Sturfelt G (1999). Synergetic effect between interleukin-1 receptor antagonist allele (IL1RN∗2) and MHC class II (DR17,DQ2) in determining susceptibility to systemic lupus erythematosus. *Lupus*.

[88] Eroglu GE, Kohler PF (2002). Familial systemic lupus erythematosus: the role of genetic and environmental factors. *Annals of the Rheumatic Diseases*.

[89] Morel L (2007). Genetics of Human Lupus Nephritis. *Seminars in Nephrology*.

[90] Deng Y, Tsao BP (2010). Genetic susceptibility to systemic lupus erythematosus in the genomic era. *Nature Reviews Rheumatology*.

[91] Han J-W, Zheng H-F, Cui Y (2009). Genome-wide association study in a Chinese Han population identifies nine new susceptibility loci for systemic lupus erythematosus. *Nature Genetics*.

[92] Harley JB, Alarcón-Riquelme ME, Criswell LA (2008). Genome-wide association scan in women with systemic lupus erythematosus identifies susceptibility variants in ITGAM, PXK, KIAA1542 and other loci. *Nature Genetics*.

[93] Hom G, Graham RR, Modrek B (2008). Association of systemic lupus erythematosus with C8orf13-BLK and ITGAM-ITGAX. *The New England Journal of Medicine*.

[94] Graham RR, Cotsapas C, Davies L (2008). Genetic variants near TNFAIP3 on 6q23 are associated with systemic lupus erythematosus. *Nature Genetics*.

[95] Barcellos LF, May SL, Ramsay PP (2009). High-density SNP screening of the major histocompatibility complex in systemic lupus erythematosus demonstrates strong evidence for independent susceptibility regions. *PLoS Genetics*.

[96] Budarf ML, Goyette P, Boucher G (2011). A targeted association study in systemic lupus erythematosus identifies multiple susceptibility alleles. *Genes and Immunity*.

[97] Danchenko N, Satia JA, Anthony MS (2006). Epidemiology of systemic lupus erythematosus: a comparison of worldwide disease burden. *Lupus*.

[98] Ruiz-Narvaez EA, Fraser PA, Palmer JR (2011). MHC region and risk of systemic lupus erythematosus in African American women. *Human Genetics*.

[99] Ramos PS, Criswell LA, Moser KL (2011). A comprehensive analysis of shared loci between systemic lupus erythematosus (SLE) and sixteen autoimmune diseases reveals limited genetic overlap. *PLoS Genetics*.

[100] Waters ST, Fu SM, Gaskin F (2001). NZM2328: a new mouse model of systemic lupus erythematosus with unique genetic susceptibility loci. *Clinical Immunology*.

[101] Wakeland EK, Wandstrat AE, Liu K, Morel L (1999). Genetic dissection of systemic lupus erythematosus. *Current Opinion in Immunology*.

[102] Kono DH, Theofilopoulos AN (2006). Genetics of SLE in mice. *Springer Seminars in Immunopathology*.

[103] Xu Z, Morel L (2010). Genetics of systemic lupus erythematosus: contributions of mouse models in the era of human genome-wide association studies. *Discovery Medicine*.

[104] Morel L, Perry D, Sang A, Yin Y, Zheng YY (2011). Murine models of systemic lupus erythematosus. *Journal of Biomedicine and Biotechnology*.

[105] Wandstrat A, Wakeland E (2001). The genetics of complex autoimmune diseases: non-MHC susceptibility genes. *Nature Immunology*.

[106] Sengupta M, Morel L (2011). Lupus at the molecular level. *Protein Cell*.

[107] Morel L, Mohan C, Yu Y (1997). Functional dissection of systemic lupus erythematosus using congenic mouse strains. *Journal of Immunology*.

[108] Kono DH, Burlingame RW, Owens DG (1994). Lupus susceptibility loci in New Zealand mice. *Proceedings of the National Academy of Sciences of the United States of America*.

[109] Mohan C, Alas E, Morel L, Yang P, Wakeland EK (1998). Genetic dissection of SLE pathogenesis. Sle1 on murine chromosome 1 leads to a selective loss of tolerance to H2A/H2B/DNA subnuclesomes. *The Journal of Clinical Investigation*.

[110] Morel L, Rudofsky UH, Longmate JA, Schiffenbauer J, Wakeland EK (1994). Polygenic control of susceptibility to murine systemic lupus erythematosus. *Immunity*.

[111] Mohan C, Morel L, Yang P, Wakeland EK (1997). Genetic dissection of systemic lupus erythematosus pathogenesis: Sle2 on murine chromosome 4 leads to B cell hyperactivity. *Journal of Immunology*.

[112] Rudofsky UH, Evans BD, Balaban SL, Mottironi VD, Gabrielsen AE (1993). Differences in expression of lupus nephritis in New Zealand Mixed H-2(z) homozygous inbred strains of mice derived from New Zealand Black and New Zealand White mice: origins and initial characterization. *Laboratory Investigation*.

[113] Morel L, Blenman KR, Croker BP, Wakeland EK (2001). The major murine systemic lupus erythematosus susceptibility locus, Sle1, is a cluster of functionally related genes. *Proceedings of the National Academy of Sciences of the United States of America*.

[114] Xu Z, Cuda CM, Croker BP (2011). The NZM2410-derived lupus susceptibility locus Sle2c1 increases Th17 polarization and induces nephritis in Fas-deficient mice. *Arthritis and Rheumatism*.

[115] Liu K, Li QZ, Yu Y (2007). Sle3 and Sle5 can independently couple with Sle1 to mediate severe lupus nephritis. *Genes and Immunity*.

[116] Cuda CM, Zeumer L, Sobel ES, Croker BP, Morel L (2010). Murine lupus susceptibility locus Sle1a requires the expression of two sub-loci to induce inflammatory T cells. *Genes and Immunity*.

[117] Xu Z, Duan B, Croker BP, Wakeland EK, Morel L (2005). Genetic dissection of the murine lupus susceptibility locus Sle2: contributions to increased peritoneal B-1a cells and lupus nephritis map to different loci. *Journal of Immunology*.

[118] Li J, Liu Y, Xie C (2005). Deficiency of type I interferon contributes to Sle2-associated component lupus phenotypes. *Arthritis and Rheumatism*.

[119] Chen Y, Cuda C, Morel L (2005). Genetic determination of T cell help in loss of tolerance to nuclear antigens. *Journal of Immunology*.

[120] Morel L, Croke BP, Blenman KR (2000). Genetic reconstitution of systemic lupus erythematosus immunopathology with polycongenic murine strains. *Proceedings of the National Academy of Sciences of the United States of America*.

[121] Subramanian S, Yim YS, Liu K, Tus K, Zhou XJ, Wakeland EK (2005). Epistatic suppression of systemic lupus erythematosus: fine mapping of Sles1 to less than 1 Mb. *Journal of Immunology*.

[122] Morel L, Tian XH, Croker BP, Wakeland EK (1999). Epistatic modifiers of autoimmunity in a murine model of lupus nephritis. *Immunity*.

[123] Vyse TJ, Rozzo SJ, Drake CG (1998). Contributions of Ea(z) and Eb(z) MHC genes to lupus susceptibility in New Zealand mice. *Journal of Immunology*.

[124] Jørgensen TN, Gubbels MR, Kotzin BL (2004). New insights into disease pathogenesis from mouse lupus genetics. *Current Opinion in Immunology*.

[125] Mozes E, Lovchik J, Zinger H, Singer DS (2005). MHC class I expression regulates susceptibility to spontaneous autoimmune disease in (NZBxNZW)F1 mice. *Lupus*.

[126] Vidal S, Kono DH, Theofilopoulos AN (1998). Loci predisposing to autoimmunity in MRL-Fas(lpr) and C57BL/6-Fas(lpr) mice. *The Journal of Clinical Investigation*.

[127] Price P, Witt C, Allcock R (1999). The genetic basis for the association of the 8.1 ancestral haplotype (A1, B8, DR3) with multiple immunopathological diseases. *Immunological Reviews*.

[128] Walport MJ, Black CM, Batchelor JR (1982). The immunogenetics of SLE. *Clinics in Rheumatic Diseases*.

[129] Ulgiati D, Abraham LJ (1996). Comparative analysis of the disease-associated complement C4 gene from the HLA-A1, B8, DR3 haplotype. *Experimental and Clinical Immunogenetics*.

[130] Bishof NA, Welch TR, Beischel LS, Carson D, Donnelly PA (1993). DP polymorphism in HLA-A1,-B8,-DR3 extended haplotypes associated with membranoproliferative glomerulonephritis and systemic lupus erythematosus. *Pediatric Nephrology*.

[131] Griffing WL, Moore SB, Luthra HS (1980). Associations of antibodies to native DNA with HLA-DRw3. A possible major histocompatibility complex-linked human immune response gene. *The Journal of Experimental Medicine*.

[132] Singer DS, Mozes E, Kirshner S, Kohn LD (1997). Role of MHC class I molecules in autoimmune disease. *Critical Reviews in Immunology*.

[133] McPhee CG, Sproule TJ, Shin D-M (2011). MHC class I family proteins retard systemic lupus erythematosus autoimmunity and B cell lymphomagenesis. *Journal of Immunology*.

[134] Chan OTM, Paliwal V, McNiff JM, Park SH, Bendelac A, Shlomchik MJ (2001). Deficiency in *β*2-microglobulin, but not CD1, accelerates spontaneous lupus skin disease while inhibiting nephritis in MRL-Faslpr mice: an example of disease regulation at the organ level. *Journal of Immunology*.

[135] Mozes E, Kohn LD, Hakim F, Singer DS (1993). Resistance of MHC class I-deficient mice to experimental systemic lupus erythematosus. *Science*.

[136] Christianson GJ, Blankenburg RL, Duffy TM (1996). *β*2-Microglobulin dependence of the lupus-like autoimmune syndrome of MRL-lpr mice. *Journal of Immunology*.

[137] Black CM, Welsh KI, Fielder A (1982). HLA antigens and Bf allotypes in SLE: evidence for the association being with specific haplotypes. *Tissue Antigens*.

[138] Celada A, Barras C, Benzonana G, Jeannet M (1980). Increased frequency of HLA-DRw3 in systemic lupus erythematosus. *Tissue Antigens*.

[139] Gladman DD, Terasaki PI, Park MS (1979). Increased frequency of HLA-DRW2 in SLE. *The Lancet*.

[140] Scherak O, Smolen JS, Mayr WR (1979). Prevalence of HLA-DRw2 not increased in systemic lupus erythematosus. *The New England Journal of Medicine*.

[141] Graham RR, Ortmann W, Rodine P (2007). Specific combinations of HLA-DR2 and DR3 class II haplotypes contribute graded risk for disease susceptibility and autoantibodies in human SLE. *European Journal of Human Genetics*.

[142] Paisansinsup T, Deshmukh US, Chowdhary VR, Luthra HS, Fu SM, David CS (2002). HLA class II influences the immune response and antibody diversification to Ro60/Sjögren’s syndrome-A: heightened antibody responses and epitope spreading in mice expressing HLA-DR molecules. *Journal of Immunology*.

[143] Jiang C, Deshmukh US, Gaskin F (2010). Differential responses to Smith D autoantigen by mice with HLA-DR and HLA-DQ transgenes: dominant responses by HLA-DR3 transgenic mice with diversification of autoantibodies to small nuclear ribonucleoprotein, double-stranded DNA, and nuclear antigens. *Journal of Immunology*.

[144] Pisetsky DS (2000). Anti-DNA and autoantibodies. *Current Opinion in Rheumatology*.

[145] Franceschini F, Cavazzana I (2005). Anti-Ro/SSA and La/SSB antibodies. *Autoimmunity*.

[146] Heward J, Gough SCL (1997). Genetic susceptibility to the development of autoimmune disease. *Clinical Science*.

[147] Hamilton RG, Harley JB, Bias WB (1988). Two Ro (SS-A) autoantibody responses in systemic lupus erythematosus: correlation of HLA-DR/DQ specificities with quantitative expression of Ro (SS-A) autoantibody. *Arthritis and Rheumatism*.

[148] Vyse TJ, Drake CG, Rozzo SJ, Roper E, Izui S, Kotzin BL (1996). Genetic linkage of IgG autoantibody production in relation to lupus nephritis in New Zealand hybrid mice. *The Journal of Clinical Investigation*.

[149] Yoshida H, Kohno A, Ohta K (1981). Genetic studies of autoimmunity in New Zealand mice. III. Associations among anti-DNA antibodies, NTA, and renal disease in (NZBxNZW)F1xNZW backcross Mice. *Journal of Immunology*.

[150] Azizah MR, Ainol SS, Kuak SH, Kong NCT, Normaznah Y, Rahim MN (2001). The association of the HLA class II antigens with clinical and autoantibody expression in Malaysian Chinese patients with systemic lupus erythematosus. *Asian Pacific Journal of Allergy and Immunology*.

[151] Hartung K, Coldewey R, Krapf F (1991). Hetero- and homozygosity of MHC class II gene products in systemic lupus erythematosus. *Tissue Antigens*.

[152] Mattos P, Santiago MB (2011). Association of antiphospholipid antibodies with valvulopathy in systemic lupus erythematosus: a systematic review. *Clinical Rheumatology*.

[153] Love PE, Santoro SA (1990). Antiphospholipid antibodies: anticardiolipin and the lupus anticoagulant in systemic lupus erythematosus (SLE) and in non-SLE disorders. Prevalence and clinical significance. *Annals of Internal Medicine*.

[154] Ruiz-Irastorza G, Crowther M, Branch W, Khamashta MA (2010). Antiphospholipid syndrome. *The Lancet*.

[155] Savi M, Ferraccioli GF, Neri TM (1988). HLA-DR antigens and anticardiolipin antibodies in Northern Italian systemic lupus erythematosus patients. *Arthritis and Rheumatism*.

[156] Azizah MR, Ainol SS, Kong NC, Normaznah Y, Rahim MN (2001). HLA antigens in Malay patients with systemic lupus erythematosus: association with clinical and autoantibody expression. *The Korean Journal of Internal Medicine*.

[157] Truedsson L, Sturfelt G, Johansen P, Nived O, Thuresson B (1995). Sharing of MHC haplotypes among patients with systemic lupus erythematosus from unrelated caucasian multicase families: disease association with the extended haplotype [HLA-B8,SCO1,DR17]. *Journal of Rheumatology*.

[158] Jönsen A, Bengtsson AA, Sturfelt G, Truedsson L (2004). Analysis of HLA DR, HLA DQ, C4A, FcgammaRIIa, FcgammaRIIIa, MBL, and IL-1Ra allelic variants in Caucasian systemic lupus erythematosus patients suggests an effect of the combined FcgammaRIIa R/R and IL-1Ra 2/2 genotypes on disease susceptibility. *Arthritis Research and Therapy*.

[159] Skarsvag S, Nansen KE, Hols A, Moen T (1992). Distribution of HLA class II alleles among Scandinavian patients with systemic lupus erythematosus (SLE): an increased risk of SLE among non[DRB1*03,DQA1*0501,DQB1*0201] class II homozygotes?. *Tissue Antigens*.

[160] Yao Z, Kimura A, Hartung K (1993). Polymorphism of the DQA1 promoter region (QAP) and DRB1, QAP, DQA1, DQB1 haplotypes in systemic lupus erythematosus. *Immunogenetics*.

[161] Doherty DG, Ireland R, Demaine AG (1992). Major histocompatibility complex genes and susceptibility to systemic lupus erythematosus in Southern Chinese. *Arthritis and Rheumatism*.

[162] Yao Z, Hartung K, Deicher HG (1993). Dna typing for HLA-DPB 1-alleles in German patients with systemic lupus erythematosus using the polymerase chain reaction and DIG-ddUTP-labelled oligonucleotide probes. *European Journal of Immunogenetics*.

[163] Fouad F, Johny K, Kaaba S, Alkarmi TO, Sharma P, Al-Harbi S (1994). MHC in systemic lupus erythematosus: a study on a Kuwaiti population. *European Journal of Immunogenetics*.

[164] Graham RR, Ortmann WA, Langefeld CD (2002). Visualizing human leukocyte antigen class II risk haplotypes in human systemic lupus erythematosus. *American Journal of Human Genetics*.

[165] Fernando MM, Stevens CR, Sabeti PC (2007). Identification of two independent risk factors for lupus within the MHC in United Kingdom families. *PLoS Genetics*.

[166] Hartung K, Baur MP, Coldewey R (1992). Major histocompatibility complex haplotypes and complement C4 alleles in systemic lupus erythematosus. Results of a multicenter study. *The Journal of Clinical Investigation*.

[167] Halloran PF, Urmson J, Ramassar V, Laskin C, Autenried P (1988). Increased class I and class II MHC products and mRNA in kidneys of MRL-1pr/1pr mice3 during autoimmune nephritis and inhibition by cyclosporine. *Journal of Immunology*.

[168] Jevnikar AM, Grusby MJ, Glimcher LH (1994). Prevention of nephritis in major histocompatibility complex class II- deficient MRL-lpr mice. *The Journal of Experimental Medicine*.

[169] Theofilopoulos AN, Dixon FJ (1981). Etiopathogenesis of murine SLE. *Immunological Reviews*.

[170] Sekine H, Graham KL, Zhao S (2006). Role of MHC-linked genes in autoantigen selection and renal disease in a murine model of systemic lupus erythematosus. *Journal of Immunology*.

[171] Howie JB, Helyer BJ (1968). The immunology and pathology of NZB mice. *Advances in Immunology*.

[172] Hirose S, Nagasawa R, Sekikawa I (1983). Enhancing effect of H-2-linked NZW gene(s) on the autoimmune traits of (NZB x NZW)F1 mice. *The Journal of Experimental Medicine*.

[173] Hirose S, Ueda G, Noguchi K (1986). Requirement of H-2 heterozygosity for autoimmunity in (NZB x NZW)F1 hybrid mice. *European Journal of Immunology*.

[174] Zhang D, Fujio K, Jiang Y (2004). Dissection of the role of MHC class II A and E genes in autoimmune susceptibility in murine lupus models with intragenic recombination. *Proceedings of the National Academy of Sciences of the United States of America*.

[175] Hirose S, Kinoshita K, Nozawa S, Nishimura H, Shirai (1990). Effects of major histocompatibility complex on autoimmune disease of H-2-congenic New Zealand mice. *International Immunology*.

[176] Murphy ED, Roths JB (1979). A Y chromosome associated factor in strain BXSB producing accelerated autoimmunity and lymphoproliferation. *Arthritis and Rheumatism*.

[177] Deane JA, Pisitkun P, Barrett RS (2007). Control of toll-like receptor 7 expression is essential to restrict autoimmunity and dendritic cell proliferation. *Immunity*.

[178] Kono DH, Haraldsson MK, Lawson BR (2009). Endosomal TLR signaling is required for anti-nucleic acid and rheumatoid factor autoantibodies in lupus. *Proceedings of the National Academy of Sciences of the United States of America*.

[179] Kelley J, Johnson MR, Alarcón GS, Kimberly RP, Edberg JC (2007). Variation in the relative copy number of the TLR7 gene in patients with systemic lupus erythematosus and healthy control subjects. *Arthritis and Rheumatism*.

[180] Shen N, Fu Q, Deng Y (2010). Sex-specific association of X-linked toll-like receptor 7 (TLR7) with male systemic lupus erythematosus. *Proceedings of the National Academy of Sciences of the United States of America*.

[181] Kawasaki A, Furukawa H, Kondo Y (2011). TLR7 single-nucleotide polymorphisms in the 3’ untranslated region and intron 2 independently contribute to systemic lupus erythematosus in Japanese women: a case-control association study. *Arthritis Research and Therapy*.

[182] García-Ortiz H, Velázquez-Cruz R, Espinosa-Rosales F, Jiménez-Morales S, Baca V, Orozco L (2010). Association of TLR7 copy number variation with susceptibility to childhood-onset systemic lupus erythematosus in Mexican population. *Annals of the Rheumatic Diseases*.

[183] Zipfel PF (2009). Complement and immune defense: from innate immunity to human diseases. *Immunology Letters*.

[184] Zipfel PF, Skerka C (2009). Complement regulators and inhibitory proteins. *Nature Reviews Immunology*.

[185] Tansey MG, Szymkowski DE (2009). The TNF superfamily in 2009: new pathways, new indications, and new drugs. *Drug Discovery Today*.

[186] Grewal IS (2009). Overview of TNF superfamily: a chest full of potential therapeutic targets. *Advances in Experimental Medicine and Biology*.

[187] Daugaard M, Rohde M, Jäättelä M (2007). The heat shock protein 70 family: highly homologous proteins with overlapping and distinct functions. *FEBS Letters*.

[188] Kudla G, Helwak A, Lipinski L (2004). Gene conversion and GC-content evolution in mammalian Hsp70. *Molecular Biology and Evolution*.

[189] Sarma JV, Ward PA (2011). The complement system. *Cell and Tissue Research*.

[190] Carroll MV, Sim RB (2011). Complement in health and disease. *Advanced Drug Delivery Reviews*.

[191] Navratil JS, Korb LC, Ahearn JM (1999). Systemic lupus erythematosus and complement deficiency: clues to a novel role for the classical complement pathway in the maintenance of immune tolerance. *Immunopharmacology*.

[192] Cook HT, Botto M (2006). Mechanisms of disease: the complement system and the pathogenesis of systemic lupus erythematosus. *Nature Clinical Practice Rheumatology*.

[193] Pickering MC, Botto M, Taylor PR, Lachmann PJ, Walport MJ (2000). Systemic lupus erythematosus, complement deficiency, and apoptosis. *Advances in Immunology*.

[194] Morgan BP, Walport MJ (1991). Complement deficiency and disease. *Immunology Today*.

[195] Colten HR (1983). Molecular genetics of the major histocompatibility linked complement genes. *Springer Seminars in Immunopathology*.

[196] Kölble K, Reid KB (1993). Genetic deficiencies of the complement system and association with disease—early components. *International Reviews of Immunology*.

[197] Glass D, Raum D, Gibson D (1976). Inherited deficiency of the second component of complement. Rheumatic disease associations. *The Journal of Clinical Investigation*.

[198] Fielder AHL, Walport MJ, Batchelor JR (1983). Family study of the major histocompatibility complex in patients with systemic lupus erythematosus: importance of null alleles of C4A and C4B in determining disease susceptibility. *BMJ*.

[199] Yu CY, Belt KT, Giles CM, Campbell RD, Porter RR (1986). Structural basis of the polymorphism of human complement components C4A and C4B: gene size, reactivity and antigenicity. *The EMBO Journal*.

[200] Mauff G, Luther B, Schneider PM (1998). Reference typing report for complement component C4. *Experimental and Clinical Immunogenetics*.

[201] Yang Y, Chung EK, Yee LW (2007). Gene copy-number variation and associated polymorphisms of complement component C4 in human systemic lupus erythematosus (SLE): low copy number is a risk factor for and high copy number is a protective factor against SLE susceptibility in European Americans. *American Journal of Human Genetics*.

[202] Olsen ML, Goldstein R, Arnett FC, Duvic M, Pollack M, Reveille JD (1989). C4A gene deletion and HLA associations in black Americans with systemic lupus erythematosus. *Immunogenetics*.

[203] Botto M, Fong KY, So AK (1992). Homozygous hereditary C3 deficiency due to a partial gene deletion. *Proceedings of the National Academy of Sciences of the United States of America*.

[204] Davies KA, Schifferli JA, Walport MJ (1994). Complement deficiency and immune complex disease. *Springer Seminars in Immunopathology*.

[205] Mũoz LE, Lauber K, Schiller M, Manfredi AA, Herrmann M (2010). The role of defective clearance of apoptotic cells in systemic autoimmunity. *Nature Reviews Rheumatology*.

[206] Carroll MC (2000). The role of complement in B cell activation and tolerance. *Advances in Immunology*.

[207] Lipsky PE (2001). Systemic lupus erythematosus: an autoimmune disease of B cell hyperactivity. *Nature Immunology*.

[208] Pattanakitsakul SN, Zheng JH, Natsuume-Sakai S, Takahashi M, Nonaka M (1992). Aberrant splicing caused by the insertion of the B2 sequence into an intron of the complement C4 gene is the basis for low C4 production in H-2(k) mice. *The Journal of Biological Chemistry*.

[209] Garlepp MJ, Hart DA, Fritzler MJ (1989). Regulation of plasma complement C4 and Factor B levels in murine systemic lupus erythematosus. *Journal of Clinical and Laboratory Immunology*.

[210] Botto M, Dell’Agnola C, Bygrave AE (1998). Homozygous C1q deficiency causes glomerulonephritis associated with multiple apoptotic bodies. *Nature Genetics*.

[211] Botto M (2001). Links between complement deficiency and apoptosis. *Arthritis Research*.

[212] Taylor PR, Carugati A, Fadok VA (2000). A hierarchical role for classical pathway complement proteins in the clearance of apoptotic cells in vivo. *The Journal of Experimental Medicine*.

[213] Ritossa F (1996). Discovery of the heat shock response. *Cell Stress and Chaperones*.

[214] Ripley BJM, Isenberg DA, Latchman DS (2001). Elevated levels of the 90 kDa heat shock protein (hsp90) in SLE correlate with levels of IL-6 and autoantibodies to hsp90. *Journal of Autoimmunity*.

[215] Walter L, Rauh F, Gunther E (1994). Comparative analysis of the three major histocompatibility complex-linked heat shock protein 70 (Hsp70) genes of the rat. *Immunogenetics*.

[216] Favatier F, Bornman L, Hightower LE, Günther E, Polla BS (1997). Variation in hsp gene expression and Hsp polymorphism: do they contribute to differential disease susceptibility and stress tolerance?. *Cell Stress and Chaperones*.

[217] Jarjour W, Reed AM, Gauthier J, Hunt S, Winfield JB (1996). The 8.5-kb PstI allele of the stress protein gene, Hsp70-2: an independent risk factor for systemic lupus erythematosus in African Americans?. *Human Immunology*.

[218] Hehlgans T, Pfeffer K (2005). The intriguing biology of the tumour necrosis factor/tumour necrosis factor receptor superfamily: players, rules and the games. *Immunology*.

[219] Cawthorn WP, Sethi JK (2008). TNF-*α* and adipocyte biology. *FEBS Letters*.

[220] Sugarman BJ, Aggarwal BB, Hass PE (1985). Recombinant human tumor necrosis factor-*α*: effects on proliferation of normal and transformed cells in vitro. *Science*.

[221] Beutler B, Cerami A (1989). The biology of cachectin/TNF—a primary mediator of the host response. *Annual Review of Immunology*.

[222] Zembala M, Kowalczyk D, Pryjma J (1990). The role of tumor necrosis factor in the regulation of antigen presentation by human monocytes. *International Immunology*.

[223] Wilson AG, Di Giovine FS, Blakemore AIF, Duff GW (1992). Single base polymorphism in the human Tumour Necrosis Factor alpha (TNF*α*) gene detectable by NcoI restriction of PCR product. *Human Molecular Genetics*.

[224] Wilson AG, Gordon C, Di Giovine FS (1994). A genetic association between systemic lupus erythematosus and tumor necrosis factor alpha. *European Journal of Immunology*.

[225] Rudwaleit M, Tikly M, Khamashta M (1996). Interethnic differences in the association of tumor necrosis factor promoter polymorphisms with systemic lupud erythematosus. *Journal of Rheumatology*.

[226] Rood MJ, Van Krugten MV, Zanelli E (2000). TNF-308A and HLA-DR3 alleles contribute independently to susceptibility to systemic lupus erythematosus. *Arthritis and Rheumatism*.

[227] Schotte H, Willeke P, Tidow N (2005). Extended haplotype analysis reveals an association of TNF polymorphisms with susceptibility to systemic lupus erythematosus beyond HLA-DR3. *Scandinavian Journal of Rheumatology*.

[228] Muller U, Jongeneel CV, Nedospasov SA (1987). Tumour necrosis factor and lymphotoxin genes map close to H-2D in the mouse major histocompatibility complex. *Nature*.

[229] Jacob CO, Hwang F, Lewis GD, Stall AM (1991). Tumor necrosis factor alpha in murine systemic lupus erythematosus disease models: implications for genetic predisposition and immune regulation. *Cytokine*.

[230] Jacob CO, McDevitt HO (1988). Tumour necrosis factor-*α* in murine autoimmune “lupus” nephritis. *Nature*.

[231] Kontoyiannis D, Kollias G (2000). Accelerated autoimmunity and lupus nephritis in NZB mice with an engineered heterozygous deficiency in tumor necrosis factor. *European Journal of Immunology*.

[232] Artzt K, Barlow D, Dove WF (1991). Mouse chromosome 17. *Mammalian Genome*.

[233] Endo T, Imanishi T, Gojobori T, Inoko H (1997). Evolutionary significance of intra-genome duplications on human chromosomes. *Gene*.

